# How Environmental Uncertainty Moderates the Effect of Relative Advantage and Perceived Credibility on the Adoption of Mobile Health Services by Chinese Organizations in the Big Data Era

**DOI:** 10.1155/2016/3618402

**Published:** 2016-12-25

**Authors:** Xing Chen, Xing Zhang

**Affiliations:** ^1^School of Public Administration, China University of Geosciences (Wuhan), Wuhan 430074, China; ^2^School of Management, Wuhan Textile University, Wuhan 430200, China

## Abstract

Despite the importance of adoption of mobile health services by an organization on the diffusion of mobile technology in the big data era, it has received minimal attention in literature. This study investigates how relative advantage and perceived credibility affect an organization's adoption of mobile health services, as well as how environmental uncertainty changes the relationship of relative advantage and perceived credibility with adoption. A research model that integrates relative advantage, perceived credibility, environmental uncertainty, and an organization's intention to use mobile health service is developed. Quantitative data are collected from senior managers and information systems managers in 320 Chinese healthcare organizations. The empirical findings show that while relative advantage and perceived credibility both have positive effects on an organization's intention to use mobile health services, relative advantage plays a more important role than perceived credibility. Moreover, environmental uncertainty positively moderates the effect of relative advantage on an organization's adoption of mobile health services. Thus, mobile health services in environments characterized with high levels of uncertainty are more likely to be adopted because of relative advantage than in environments with low levels of uncertainty.

## 1. Introduction

A big data revolution has taken place in the field of healthcare. The availability of big data in healthcare advances the changing models of treatment delivery, real-time detection of diseases, prediction of epidemics, improvement of the quality of life, and development of healthcare [1]. However, acquisition and sharing of health data is difficult and costly in a traditional system [2]. With the substantial improvement in the processing capabilities of smart phones, many mobile applications can be used to collect personal health information to be adopted as big data [3]. Rapid developments of wearable devices and big data technologies have led the health services to the era of mobile health. Mobile health is broadly defined as “healthcare to anyone, anytime, and anywhere by removing locational and temporal constraints while increasing both the coverage and the quality of healthcare” [4]. Mobile health services (MHS) can be classified into five types: (1) health information retrieval; (2) remote reservation; (3) remote diagnosis; (4) electronic medical records access; (5) health consultation [5]. MHS facilitate healthcare activities for individuals and organizations anytime and anywhere [6, 7]. Through MHS, individuals can obtain relevant medical health service and information, actively participate in health management, and aim at prevention instead of treatment [8]. Organizations can use MHS to improve healthcare monitoring and alerting systems, collect and maintain clinical data, optimize the diagnosis process, and detect drug counterfeiting [9]. Mobile health services have been adopted for a wide range of industries. It has been predicted that the global market value of mobile health service will achieve a value of 97 billion dollars in 2016 [10]. With the increasing number of mobile users in China, mobile health service has received increasing attention. In 2013, there were approximately 500 million mobile phone users in China and with the advent of 4G and the large dependence on mobile health services, the Chinese mobile health service market value has achieved a value of 106 billion RMB, and it is estimated to reach 600 billion RMB in 2017 [11].

Prior studies explored the factors that affect the adoption of MHS by individuals. Consequently, several theories have been put forward to explain the adoption [12, 13]. Surprisingly, understanding the ways in which organizations adopt MHS and the factors that drive the intention of organizations to use MHS remain unclear. Two-factor theory suggests that factors influencing the user intention can be divided into motivation and hygiene factors [14]. However, previous studies have seldom investigated motivation and hygiene factors simultaneously in examining MHS adoption at the organizational level. The current study classifies relative advantage and perceived credibility, which influence the intention of organizations to use MHS, into motivation and hygiene factors. On one hand, relative advantage is the degree to which MHS is perceived as better than the idea that it supersedes. On the other hand, perceived credibility is the extent to which an organization believes that using MHS is free from security and privacy threats. Studies that prove the effect of these factors on the adoption by individual users are limited [15, 16]. By contrast, few data can show the relationship of relative advantage and perceived credibility with the intention to use mobile health services (IUMHS) at the organizational level. Therefore, the first objective of this study is to investigate the ways in which relative advantage and perceived credibility affect an organization's IUMHS.

Previous studies have examined various determinants of adoption [17, 18]. Among these however, few studies have investigated which determinant is more important relative to the other determinants. Understanding the issue is significant because mobile health service providers can enhance the most critical factor that affects the adoption of mobile health services. Based on these, the second objective of the present study is to determine the importance of relative advantage and perceived credibility on an organization's IUMHS.

In addition to the internal factors (e.g., relative advantage and perceived credibility) that may influence the intention to use mobile health services, external factors may also affect the adoption by organizations [19]. An integrated analysis of the effects of external factors on the internal mechanism of an organization is necessary. Based on the contingency theory, environmental characteristics have been found to be the potential moderators [20, 21]. In the present study, we focus on environmental uncertainty, which refers to the uncertainty around an organization [22]. Environmental uncertainty has been widely used as a moderator in previous research [23]. It has also received great attention by both researchers and senior managers because it is an unpredictable factor [24, 25]. Therefore, our third objective is to examine how environmental uncertainty changes the relationship of relative advantage and perceived credibility with the intention to use mobile health services by organizations.

The remainder of the paper is organized as follows. First, we introduce the theoretical background, the research model, and our hypotheses. Second, we describe the methodology for testing the hypotheses based on the quantitative data collected from 320 Chinese healthcare organizations. Finally, we present the results and summarize the implications for both research and practice.

## 2. Theoretical Background, Research Model, and Hypothesis Development

### 2.1. Two-Factor Theory and Organizational Adoption of Mobile Health Services

Two-factor theory, which is also known as “hygiene-motivation theory,” is one of the motivation theories [26]. It was initially used to explain employees' job satisfaction. Recently, some researchers have adapted this theory and applied it to various research contexts [14, 27, 28]. In the information systems (IS) research context, researchers classified two factors into motivation and hygiene factors. Motivation factors refer to the enablers that stimulate users to adopt a technology, whereas hygiene factors are the barriers that inhibit users from adopting such technology [14]. As motivation factors is not simply the opposite of hygiene factors [28]; both motivation and hygiene factors should be included in the current study to better understand the intention of organizations to adopt MHS.

Several motivation and hygiene factors that affect user's adoption of MHS have been identified, as shown in Table 1. Prior studies on MHS adoption focused on explaining the adoption processes at the individual level but rarely at the organizational level [29]. Factors that affect the MHS adoption of organizations are similar to that of an individual; however, the former is distinct in terms of motivators and demotivators of the adoption. On one hand, organizations aim for different benefits in using MHS. Individuals use MHS to improve their behavior, lifestyles, and work patterns, thereby enhancing their physical quality [17]. Thus, personal outcome related to health improvement (e.g., perceived value, perceived ease of use, enjoyment, and perceived usefulness) are strongly emphasized for the individual user's adoption [7, 18, 19]. However, these motivators may be not the main drivers for the adoption of MHS by organizations. MHS can create value for the organization because they improve the flexibility of organizations [30] and help in the development of new business models, new methods in their services, and business solutions [31]. These results make organizations become more innovative and gain a competitive edge.

On the other hand, individuals' resistance to MHS has always been attributed to concerns about their inadequate capacity and significant effort in learning about new technology. Thus, some personal capacity factors (e.g., self-efficacy, technology anxiety, perceived physical condition, and resistance to change) and technological factors (e.g., technological conditions, quality, and compatibility) are hygiene factors that have been extensively investigated in previous MHS adoption studies. However, organizations may worry about the ways in which their information privacy can be protected while using this new technology. They demand for confidentiality and privacy issues. From the above discussion, we can infer that organizations have different views on the benefits and risks of adopting MHS. How to gain relative advantage and reduce privacy concerns are burning concerns when introducing MHS to an organization. We then investigate relative advantage and perceived credibility as two antecedents of organization's IUMHS.

### 2.2. Relative Advantage

Among the adoption theories, the innovation diffusion theory developed by Rogers [32] is frequently cited by researchers who examined adoption and diffusion of information systems and information technology related services [33, 34]. Rogers established the relationship between perceived innovation attributes (i.e., relative advantage, compatibility, complexity, trialability, and observability) and the rate of adoption [32]. The theory is regarded as important in predicting the adoption of technological innovations such as mobile health services.

Based on prior research [35, 36], relative advantage is defined as the degree with which the mobile health service is perceived as better than the idea that it supersedes. For individual users, improving the relative advantage may include improving the quality of work, increasing the speed of accomplishing task, and enhancing the effectiveness on the job by using mobile health services [36, 37]. However, for organizations, relative advantage is associated with competitiveness. The improvement of relative advantage includes improving the efficiency and profitability and reducing the operational cost of an organization. We selected relative advantage from the five innovation attributes of Rogers in [32] because relative advantage is regarded as one of the best predictors for an innovation's usage [38].

According to Rogers [32], the relative advantage of an innovation is positively related to adoption. Relative advantage exerts positive influence on the adoption of mobile Internet [36]. If mobile health services are perceived to largely improve the efficiency and profitability and strengthen the competitiveness of organizations, mobile health services will be easily accepted and reliant on. Therefore, the higher the relative advantage of the mobile health service, the higher the probability that an organization will use the mobile health services.

(H1): Relative advantage has a positive effect on an organization's IUMHS.

### 2.3. Perceived Credibility

Luarn and Lin examined the relationship between perceived credibility and user's adoption behavior [39]. However, the generalizability of their adoption model to the overall mobile health service adoption has never been investigated. In addition, their model was tested based on 180 individual users. Thus, the generalizability of the model to organizations is also unknown. Therefore, in this study, we tested the effect of perceived credibility to see if the model can explain an organization's adoption of mobile health services.

While relative advantage can be an important factor for organizations in relation to the use of mobile health services, the security and privacy concerns related to perceived credibility may also affect the adoption [39]. Based on the definition of perceived credibility for individual users [40, 41], it is defined in relation to an organization as the extent to which an organization believes that using mobile health services will ensure freedom from security and privacy threats.

Perceived credibility was found to have a significant positive effect on the adoption of different kinds of mobile health services [39, 40, 42]. Credibility enhances the confidence and trust of organizations to use mobile health services. Mobile health services also receive greater trust by organizational users and thus increase the intention to use them if the credibility of mobile health services is perceived to be high [43]. Organizations become less likely to use mobile health services with low perceived credibility. Thus, we posit the following hypothesis:

(H2): Perceived credibility has a positive effect on an organization's IUMHS.

### 2.4. Relative Importance of Relative Advantage and Perceived Credibility

For organizations, perceived credibility appears to be a hygiene factor, because if mobile health services are not perceived to be creditable, the organization is unlikely to use it and if mobile health services are perceived to be creditable, the adoption will slightly increase [39]. Relative advantage appears to be a motivator because if an organization can gain relative advantage from mobile health services, organizations will use mobile health services to a larger extent [32]. Based on the two-factor theory, motivators are more important than hygiene factors when both factors are resolved [26, 44]. Therefore, relative advantage will be a more important factor than perceived credibility in relation to the use of mobile health services by organizations. Thus, we posit the following hypothesis:

(H3): The relationship between relative advantage and an organization's IUMHS is stronger than the relationship between perceived credibility and the organization IUMHS.

### 2.5. Moderating Role of Environmental Uncertainty

Environmental uncertainty, which can be defined as the uncertainty around an organization, comprises three aspects, namely, dynamism, heterogeneity, and hostility [45, 46]. Dynamism reflects the unpredictable changes in the demands and behaviors of competitors. Heterogeneity reflects the diversity of customer behavior and products or services. Hostility reflects the degree of competition in a competitive environment. Based on information processing theory [47], an uncertain environment requires an organization to possess a higher information processing ability. Mobile health services can enable organizations to obtain and process critical information anytime and anywhere [48], which can magnify the effect of the relative advantage on IUMHS. Moreover, in environments with high levels of uncertainty, competition increases the rate of innovation adoption [49]. By using innovative information technology, the competitive ability of the organization can be significantly enhanced [50]. First, the use of MHS can alter the methods of delivering medical services, thereby changing the industrial structure and the rules of the competition. Second, MHS can also create a competitive advantage by helping organizations obtain customers' requirements more quickly and efficiently. Finally, MHS will bring organizations with new health services, which will help them to outperform their competitors. Therefore, organizations in an environment of with a high level of uncertainty would feel a greater need to use MHS to gain a competitive advantage. Thus, we propose the following hypothesis:

(H4): The positive effect of relative advantage and IUMHS is moderated by environmental uncertainty such that the positive effect is greater when environmental uncertainty is high.

High environmental uncertainty requires organizations to use more creditable technology and management approaches to gain competitive advantage [22]. This can amplify the impact of perceived credibility on an organization's adoption of mobile health services. Moreover, the adoption of information technology will cause anxiety and discomfort to the users [51]. The information technology needs evaluation before being adopted by organizations so as to meet more information processing requirements under conditions of environmental uncertainty [52, 53]. Therefore, more perceived credibility is required for a reduction in uncertainty when organizations decide to adopt a MHS. Thus, we propose the following hypothesis:

(H5): The positive effect of perceived credibility and IUMHS is moderated by environmental uncertainty such that the positive effect is greater when environmental uncertainty is high.

### 2.6. Control Variables

The influence of three control variables is studied to test their influence on organizations' IUMHS. These variables are industry, organization size, and organization age. First, organizations in different industries may adopt mobile health services differently. Second, large organizations may have higher intention to use mobile health services because a significant number of users can obtain the value of mobile health services. Third, IUMHS may also differ between old and young organizations because mobile health services are relatively new technology and may be better accepted by developing organizations. Therefore, we control the effect of these three variables.

**Figure 1:**
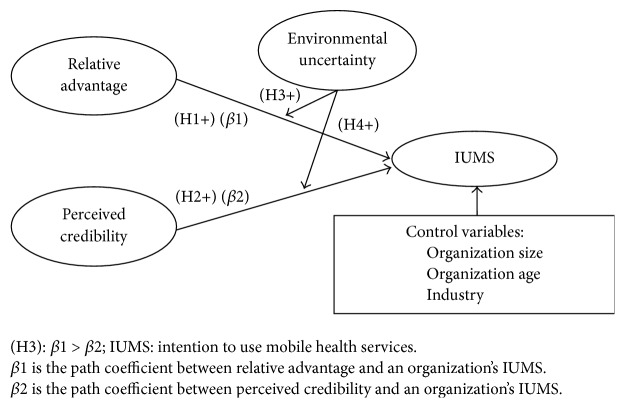
Research model.

Based on the above analysis, we formulate the research model and present it in Figure 1.

## 3. Research Methodology

### 3.1. Data Collection

With the assistance of a Chinese research center, we obtained a list of 500 Chinese organizations in the health sector. Senior executives and information systems (IS) managers of these organizations were selected as respondents. They were contacted to determine their willingness to participate in the survey. Senior managers are defined as those who lead an organization and supervise the main business department. They were asked to provide the basic information of their organizations and respond to the questions related to relative advantage, perceived credibility, and environmental uncertainty because they have a comprehensive understanding of their organization. IS managers are defined as those who lead the IS department of the organization and are responsible for IT management in their organizations. They were requested to respond to the questions related to IUMHS because they are key decision makers who could determine whether or not mobile health services can be adopted by their organization.

Questionnaires were mailed to the senior managers and IS managers of the selected organizations. After four months, a total of 320 usable questionnaires were returned. The response rate was 64%. The ages of senior managers range from 23 to 52; 192 of the senior managers are male and 128 are female. The ages of IS managers range from 20 to 48; 215 of them are male and 105 are female. The characteristics of the selected organizations are summarized in Table 2.

### 3.2. Construct Measurement

In the research model, measures for each construct are adapted from existing measures in literature. Five-point likert-type scales ranging from “strongly disagree” to “strongly agree” were used for the items of each construct. A pretest was conducted on 20 managers. Questionnaires were modified based on their feedback. All constructs and measures and their sources are shown in Table 3.

## 4. Results

### 4.1. Measurement Model

We used partial least squares (PLS) for data analysis. PLS is appropriate for small samples and can obtain considerable explained variances. Smart PLS 2.0 was used to evaluate the measurement model. Each construct of the measurement model was formulated to be reflective [66].

Convergent validity of all constructs was evaluated by examining cross loadings, composite reliability, and average variance extracted (AVE).  Table 4 shows that all item-to-construct loadings are greater than 0.70, indicating good internal consistency [67, 68]. The means and standard deviations of constructs, construct correlations, composite reliabilities, Cronbach's *α*, and AVE are presented in Table 5. The values of composite reliabilities and Cronbach's *α* are higher than the threshold of 0.70 [69, 70]. The values of AVE are all higher than 0.50 [71, 72].

Discriminant validity was evaluated by examining if the squared correlation between two latent variables is less than the AVE associated with each variable [73]. As shown in Table 5, discriminant validity is satisfied. We also conducted the Harman one-factor test as suggested by [74, 75] to examine if a common method bias exists. The factor analysis for both independent and dependent variables revealed that no single factor could account for the majority of covariance. Therefore, no common method bias exists. In summary, these results provided solid evidence of the good measurement properties for the measurement model.

### 4.2. Hypotheses Testing

Research hypotheses were tested by using hierarchical regression analysis with PLS. Three models were run in PLS with the hierarchical procedure. We evaluated the effect of control variables in model 1. Subsequently, we examined the main effect model by evaluating the effect of relative advantage and perceived credibility on IUMHS in model 2. (H1) and (H2) were evaluated by model 2a and the results were used as the basis for testing (H3). Finally, we examined the moderating effect by integrating moderators, independent variables, and their interactions in model 3 ((H4) and (H5)). By comparing each model, we obtained the incremental explained variance. Bootstrap analysis was conducted with 320 respondents. The results of path coefficients, incremental changes in *R*^2^, and the *F* hierarchical value between each model are shown in Table 6.

As shown in Table 6 (model 1), two control variables (i.e., organization age and industry) were found to significantly affect IUMHS. The results suggest that an organization's IUMHS vary across different industries. Furthermore, organization age has a negative effect on IUMHS, implying that developing organizations manifest higher intentions to use mobile health services. However, organization size affects IUMHS insignificantly.

Table 6 (model 2a) shows that relative advantage significantly affects IUMHS (H1). The finding suggests that relative advantage is an important driving factor for an organization's adoption of mobile health services. If the relative advantage of certain mobile health service is perceived to be high, organizations are more likely to strengthen the adoption of the service. Moreover, perceived credibility was found to have a positive effect on an organization's adoption of mobile health service (H2). The finding indicates that perceived credibility is also an important factor for an organization's IUMHS.

Both relative advantage and perceived credibility have positive effects on IUMHS. The effect of relative advantage seems to be greater than that of perceived credibility. In order to test (H3) statistically, we compared path coefficients by using the *t*-test as proposed by previous studies [76–78]. These two path coefficients were found to be significantly different (*t* = 3.67). Therefore, (H3) is supported.

As shown in Table 6 (Models 3a), the interaction terms with positive and significant coefficients between environmental uncertainty and relative advantage (*β* = 0.08) indicate significant influences on IUMHS. The interaction term increased by 1.4% based on the value of the explained variance. The value of *F* hierarchical likewise indicates that changes in explained variance are significant. Thus, (H4) is supported. In model 3b, the interaction term with insignificant coefficient between environmental uncertainty and perceived credibility (*β* = −0.03) indicates insignificant effects on IUMHS. The interaction term increased by only 0.1% of the explained variance. Thus, (H5) is not supported. Model 3c provides further evidence on the integration of all moderators and independent variables.

The results of the hypothesis testing are summarized in Table 7. Except for (H5), all hypotheses ((H1), (H2), (H3), and (H4)) are supported.

## 5. Discussions and Implications

### 5.1. Theoretical Implications

This study has several implications for mobile health service researchers and information security researchers. For mobile health service researchers, this study represents an important step toward understanding an organization's adoption of mobile health services. Based on two-factor theory, the present study demonstrates that relative advantage and perceived credibility can explain a significant amount of variance in an organization's IUMHS. Relative advantage was also found to have a positive effect on the individual adoption of different kinds of mobile health services such as multimedia message service [36]. Thus, the result is consistent with prior findings. Future investigations can extend the research model and explore more factors that affect an organization's adoption of mobile health services.

For information security researchers, this study highlights the role of perceived credibility in an organization's adoption of mobile health services, which has been overlooked in the mobile health service literature. Perceived credibility was found to have a positive effect on an organization's IUMHS. Security over the mobile platform for mobile transactions is critical [79]. Failure to provide a secure system for mobile health services will negatively influence the adoption [80]. Perceived credibility, which indicates a high level of security, exerts positive effect on an organization's adoption of mobile health services. Moreover, the result has two implications. First, if transactions provided by mobile health services are not perceived to be secure by an organization, IUMHS will be lower. Second, more efforts should be focused on the strategies that can improve the security in the mobile health service environment to ensure that the usage of mobile health services by organizations can be more reliable.

Another significant contribution of this study is the finding that shows the higher importance of relative advantage than perceived credibility to an organization's adoption of mobile health services. This is consistent with the finding of Hsu et al. [36] that reveals the significant effect of the relative advantage on the decision of innovators/early-adopters, early-majority, and late/majority groups to use multimedia message services. By contrast, other factors affect the adoption of one or two of these groups. Moreover, the finding is consistent with the proposition that relative advantage is one of the best predictors of the adoption of an innovation [32]. Therefore, relative advantage, because of its higher effectiveness, is more important than perceived credibility in relation to the adoption of mobile health services by organizations.

The empirical results also highlight the moderating role of environmental uncertainty. Specifically, environmental uncertainty positively moderates the effect of relative advantage on IUMHS, whereas the moderating effect on the relationship between perceived credibility and IUMHS is insignificant. Therefore, the relative advantage of mobile health service can facilitate the use of mobile health service by an organization in highly uncertain environments. However, the effect of perceived credibility on IUMHS does not change in either high or low level of uncertainty. One explanation is that perceived credibility is a basic requirement for an organization to adopt mobile health services. Whether the environment is certain or uncertain cannot change the necessity of the credibility of mobile health services.

### 5.2. Practical Implications

Both relative advantage and perceived credibility play important roles in an organization's adoption of mobile health services. Therefore, while evaluating the relative advantage involved in the adoption of mobile health services, decision makers in an organization should also carefully assess the credibility and security of mobile transactions. If the credibility of mobile health services is not perceived to be high, risks on mobile transactions will emerge. Moreover, other relevant parties (e.g., mobile operator, mobile health service provider) should improve the maturation of technology and overcome security and privacy problems.

Furthermore, as relative advantage was found to be more effective than perceived credibility, organizations should select mobile health services that can strengthen their advantages. Mobile health service providers should focus not only on mobile technology, but also on the services that are beneficial to an organization.

In addition, as environmental uncertainty can strengthen the effect of relative advantage on an organization's adoption of mobile health service, managers should evaluate the environment uncertainty that their organization is engaged in. Mobile health services in an environment with high levels of uncertainty are more likely to be adopted because of relative advantage than that in environments with low levels of uncertainty.

## 6. Conclusions

This study is one of the first attempts to combine adoption theories and the contingency theory in the context of mobile health services. The findings indicate that environmental uncertainty positively moderates the effect of relative advantage on an organization's adoption of mobile health services. Another critical contribution is the comparison we made on the influence of relative advantage and perceived credibility on an organization's adoption of mobile health services. The study demonstrates that relative advantage plays a more important role than perceived credibility in the decision of organizations to use mobile health services. The third contribution is the comprehensive understanding that the study offers with regard to how relative advantage and perceived credibility affect an organization's adoption of mobile health services. Specifically, both relative advantage and perceived credibility positively affect an organization's IUMHS.

Nevertheless, this study has several limitations. First, the survey data were collected from China. People in different countries may have different perceptions of credibility. For example, people in countries with high risk avoidance may perceive the effect of credibility as insignificant to an organization's adoption of mobile health services. Second, the results are based on a limited sample size. However, the use of pair design in the sample can largely reduce the negative effects of small sample size.

Based on the results and the limitations, several directions for future research are put forward. First, future research should explore other factors (e.g., perceived usefulness, perceived ease of use) that possibly affect an organization's adoption of mobile health services based on other theories. We examined relative advantage and perceived credibility because these two factors are important not only to an organization's adoption of mobile health services, but also to other innovations. Second, another extension of our study is the examination of how relative advantage and perceived credibility affect an organization's adoption of mobile health service in other countries. Third, future studies can investigate some other motivations and other hygiene factors that can explain the intention to adopt a MHS. Finally, other factors, such as risk and age [17, 81, 82], may negatively moderate the effect of antecedents on an organization's adoption of mobile health services and should also be investigated.

## Figures and Tables

**Table 1:** Representative sampling of previous studies on MHS adoption.

Previous studies	Perspective	Motivations	Hygiene factors	Theories
[54]	Healthcare professional	Perceived usefulness, tech support and training	Compatibility, MHS self-efficacy, perceived ease of use	Revised technology acceptance model

[55]	Customers	Personalization concern	Privacy concern, trust	Privacy calculus

[56]	Hospital's professionals	Perceived usefulness, personal Innovativeness in IT, subjective norm	Perceived behavioral control, perceived ease of use	The theory of reasoned action and theory of planned behavior

[57]	Elderly people	Perceived usefulness	Perceived ease of use, resistance to change, technology anxiety, dispositional resistance to change	Dual factor model of technology acceptance

[58]	Customers	Response efficacy, subjective norm, perceived vulnerability, perceived severity	Perceived ease of use, response cost, self-efficacy	Unified theory of acceptance and use of technology

[17]	Citizens	Perceived value, subject norm, self-actualization need	Perceived behavior control, perceived physical condition, resistant to change technology anxiety	Value attitude behavior model and theory of planned behavior

[59]	Diabetic patients	Perceived usefulness	Perceived ease of use, perceived compatibility, perceived reliability, perceived privacy and Security	Revised technology acceptance model

[5]	Customers	Facilitating conditions, subjective norms		Modified theory of reasoned action

[60]	Customers	Perceived vulnerability, perceived severity	Response efficacy, self-efficacy	Protection motivation theory

[61]	Young adults	Perceived usefulness, social influence	Perceived ease of use, perceived self-efficacy, trust in the application's security, task-technology fit	Revised technology acceptance model

[62]	Citizens	Performance expectancy, social influence, facilitating conditions, hedonic motivation, waiting time	Effort expectancy, price value	Unified theory of acceptance and use of technology

**Table 2:** Organizational characteristics.

	Range	Number	Percent
Number of employee	<100	92	28.8%
	100–500	70	21.9%
	500–2000	52	16.3%
	2000–10000	72	22.5%
	>10000	34	10.6%
	*Total*	*320*	*100.0%*

Age	1 year to 5 years	52	16.3%
	6 years to 10 years	56	17.5%
	11 years to 15 years	28	8.8%
	16 to 20 years	88	27.5%
	>20 years	96	30.0%
	*Total*	*320*	*100.0%*

**Table 3:** Constructs and measures.

Construct	Item #	Measure	References
Relative advantage (RA)	RA1	Mobile health services can strengthen the competitive advantage of my organization	[32, 38, 63]
	RA2	Mobile health services can strengthen the relationship between the customers and my organization	
	RA3	Mobile health services can improve the organizational efficiency	
	RA4	Mobile health services can reduce the operational cost in my organization	
	RA5	Mobile health services can enhance my organization's prestige	

Perceived credibility (PC)	PC1	Mobile health services will not divulge my organization's private information	[39, 41]
	PC2	It is secure for my organization to conduct business transactions by using mobile health services	

Environmental uncertainty (EU)	EU1	In our industry, the technology of products or services changes quickly	[23, 64]
	EU2	Our industry has tough competition in terms of the quality or price of products or services	
	EU3	Our industry has considerable diversity with regard to competition	

Intention to use mobile health services (IUMHS)	IUMHS1	My organization has a high intention to use mobile health services	[17, 65]
	IUMHS2	My organization intends to learn about using mobile health services	
	IUMHS3	My organization plans to use mobile health services	
	IUMHS4	My organization prefers mobile health services over other types of services	

**Table 4:** The item-to-construct correlations.

	RA	PC	EU	IUMHS
RA1	**0.84 **	0.33	0.25	0.42
RA2	**0.87 **	0.30	0.31	0.50
RA3	**0.88 **	0.25	0.32	0.53
RA4	**0.86 **	0.28	0.29	0.51
RA5	**0.79 **	0.17	0.23	0.42
PC1	0.27	**0.90 **	0.25	0.35
PC2	0.30	**0.91 **	0.27	0.36
EU1	0.25	0.15	**0.78 **	0.28
EU2	0.33	0.28	**0.97 **	0.39
EU3	0.27	0.29	**0.82 **	0.31
IUMHS1	0.50	0.36	0.33	**0.91 **
IUMHS2	0.53	0.35	0.37	**0.92 **
IUMHS3	0.50	0.36	0.33	**0.91 **
IUMHS4	0.53	0.35	0.37	**0.92 **

**Table 5:** Descriptive statistics, correlations, and reliability.

	Mean	SD	Cronbach's *α*	RA	PC	EU	IUMHS
RA	2.37	0.92	0.90	CR = 0.93			
				AVE = 0.72			
PC	2.51	0.96	0.78	0.31^*∗∗*^	CR = 0.90		
					AVE = 0.82		
EU	2.45	1.13	0.81	0.13	0.09	CR = 0.89	
						AVE = 0.74	
IUMHS	2.70	0.83	0.93	0.57^*∗∗*^	0.39^*∗∗*^	0.38^*∗∗*^	CR = 0.95
							AVE = 0.83

Note: *∗∗* indicates significance at the 0.01 level.

**Table 6:** Results of hierarchical regression analysis.

	Model 1	Model 2a	Model 2b	Model 3a	Model 3b	Model 3c
Block 1: control variables						
Organization size	0.05	0.02	0.05	0.04	0.04	0.03
Organization age	−0.19^**∗∗**^	0.14^**∗∗**^	0.14^**∗∗**^	0.14^**∗∗**^	0.14^**∗∗**^	0.14^**∗∗**^
Block 2: main effects						
Relative advantage		0.52^**∗∗**^	0.46^**∗∗**^	0.47^**∗∗**^	0.46^**∗∗**^	0.47^**∗∗**^
Perceived credibility		0.31^**∗∗**^	0.27^**∗∗**^	0.27^**∗∗**^	0.27^**∗∗**^	0.27^**∗∗**^
Environmental uncertainty			0.18^**∗∗**^	0.17^**∗∗**^	0.18^**∗∗**^	0.17^**∗∗**^
Block 3: moderating effects						
Relative advantage × environmental uncertainty				0.08^**∗**^		0.11^**∗**^
Perceived credibility × environmental uncertainty					−0.03	-0.07
Δ*R*^2^ (IUMHS)		0.348	0.027	0.014	0.001	0.045
*f*^2^ (effect size)		0.574	0.047	0.025	0.002	0.078
*R*^2^ (IUMHS)	0.046	0.394	0.421	0.435	0.422	0.439
*F* hierarchical		180.317^**∗∗**^	14.596^**∗∗**^	7.731^**∗∗**^	0.540	24.171^**∗∗**^

Note: *∗* and *∗∗* indicate significance at the 0.05 and 0.01 level, respectively. One-tailed *t*-test was performed as the direction of differences was hypothesized.

**Table 7:** Results of hypothesis testing.

Hypothesis	Result
(H1): Relative advantage → IUMHS	Support
(H2): Perceived credibility → IUMHS	Support
(H3): Relative advantage > perceived credibility	Support
(H4): Relative advantage × environmental uncertainty → IUMHS	Support
(H5): Perceived credibility × environmental uncertainty → IUMHS	No support
